# Breakdown of Filamentous Myofibrils by the UPS–Step by Step

**DOI:** 10.3390/biom11010110

**Published:** 2021-01-15

**Authors:** Dina Aweida, Shenhav Cohen

**Affiliations:** Faculty of Biology, Technion Institute of Technology, Haifa 32000, Israel; dinaaweida@gmail.com

**Keywords:** ubiquitin, proteasome, autophagy, muscle atrophy, intermediate filaments, myofibrils, AAA-ATPases

## Abstract

Protein degradation maintains cellular integrity by regulating virtually all biological processes, whereas impaired proteolysis perturbs protein quality control, and often leads to human disease. Two major proteolytic systems are responsible for protein breakdown in all cells: autophagy, which facilitates the loss of organelles, protein aggregates, and cell surface proteins; and the ubiquitin-proteasome system (UPS), which promotes degradation of mainly soluble proteins. Recent findings indicate that more complex protein structures, such as filamentous assemblies, which are not accessible to the catalytic core of the proteasome in vitro, can be efficiently degraded by this proteolytic machinery in systemic catabolic states in vivo. Mechanisms that loosen the filamentous structure seem to be activated first, hence increasing the accessibility of protein constituents to the UPS. In this review, we will discuss the mechanisms underlying the disassembly and loss of the intricate insoluble filamentous myofibrils, which are responsible for muscle contraction, and whose degradation by the UPS causes weakness and disability in aging and disease. Several lines of evidence indicate that myofibril breakdown occurs in a strictly ordered and controlled manner, and the function of AAA-ATPases is crucial for their disassembly and loss.

## 1. Introduction

Proteolysis promotes tissue homeostasis by controlling protein abundance in response to extracellular and intracellular cues, and by preventing accumulation of misfolded or damaged proteins [1]. Conversely, unbalanced protein breakdown can lead to tissue wasting, accumulation of abnormal proteins, and disease (e.g., neurodegenerative diseases such as Alzheimer’s, Parkinson’s, and Huntington disease [2]). The major proteolytic system responsible for degradation of soluble proteins is the ubiquitin-proteasome system (UPS) [3], while autophagy generally facilitates the loss of organelles, and cell surface or aggregated proteins. The vast majority of soluble proteins destined for degradation by the proteasome are initially tagged by the covalent attachment of ubiquitin moieties [4,5]. In addition, intricate filamentous structures, such as the myofibrillar apparatus in striated muscles, are also ubiquitinated and efficiently degraded by the UPS in vivo, although they seem resistant to this proteolytic machinery in vitro [6]. The mechanism for myofibril breakdown has long been a mystery, and recent evidence indicates that a tightly regulated solubilization process is required for efficient degradation. The loss of this fundamental contractile machinery involves an initial degradation of the cytoskeletal networks and structural and regulatory proteins that stabilize the myofibril. Here, we summarize the mechanisms for myofibril breakdown, providing an insightful view on the role of the UPS in promoting an ordered degradation of complex filamentous structures.

## 2. Myofibrils are an Intricate Filamentous Structure

**Figure 1:**
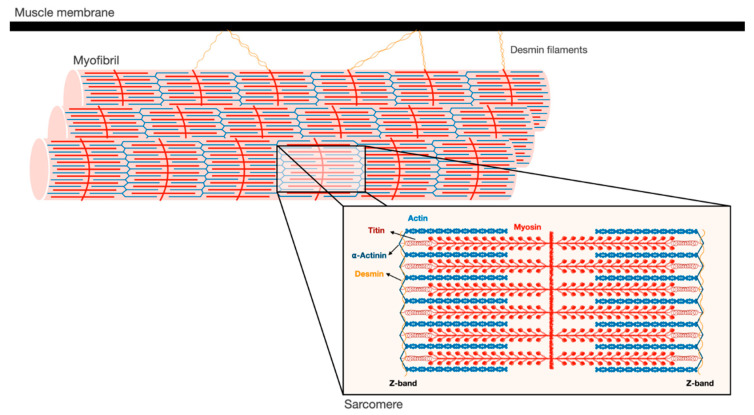
Schematic illustration of the myofibrillar apparatus. Myofibrils are a highly ordered filament system, organized in repeating units of sarcomeres. The Z-bands are the boundaries of the sarcomere and the sites where the actin thin filaments and desmin intermediate filaments are bound. Myosin thick filaments span the center of the sarcomere, and slide over actin filaments toward the Z-bands when the muscle contracts. These filaments are stabilized by additional structural proteins, such as α-actinin, which cross-links actin and desmin at the Z-bands. In addition, desmin intermediate filaments contribute to muscle architecture and contractile function by linking adjacent myofibrils laterally, and to the muscle membrane and cellular organelles.

Skeletal muscles support the skeleton and promote motion by contraction. They are composed of a bundle of long multinuclear cells, whose volume is mainly occupied by a precisely aligned filament system of myofibrils that is responsible for force production [7]. This contractile machinery is organized in repeated units of sarcomeres, which are delimited by Z-bands, and mainly contain myosin in thick, and actin in thin, filaments (Figure 1). The Myosin molecule is composed of two heavy chains (MyHC), and two pairs of regulatory (MyLC2) and essential (MyLC1) light chains, and is divided into three main regions: (1) the N-terminal head domain, which contains the motor domain that binds actin and hydrolyzes ATP to generate force; (2) the C-terminal α-helical tail domain, that constitutes the backbone of the thick filaments; and (3) the neck domain, which links the head and tail domains, and represents the site where MyLCs non-covalently bind MyHC [8]. Myosin thick filaments are stabilized at the center of the sarcomere by additional structural and regulatory proteins. For example, MyLCs, and myosin-binding protein C (MyBP-C), which binds the thick filament periodically, are required for myofibril stability and normal contractility [9,10,11]. In addition, ubiquitin ligases, proteases (e.g., calpain-3), and kinases bind myosin and regulate its activity [12].

The Z-bands constitute the anchoring site for actin thin filaments, as well as for two other filament systems in the myofibril, titin and nebulin. The thin filaments form a helical arrangement, and the protein tropomyosin lies in the α-helical grooves that are formed along the filaments. Aligned at intervals along the actin filaments is the protein complex, troponin (Tn), which is composed of three components Tn-T, Tn-I, and Tn-C, and represents the on-off switch of muscle contraction [7]. Muscle contraction is activated by Ca^2+^ influx into the cytosol, which in turn binds Tn-C, causing displacement of Tn-T and Tn-I from tropomyosin, and consequently leading to exposure of myosin binding sites on actin. Then, myosin heads bind actin and move toward the Z-bands by converting the chemical energy produced by ATP hydrolysis into mechanical force. The sliding of the thick over the thin filaments in their overlapping regions causes shortening of the sarcomeres and the muscle fiber, and powers contraction.

At the Z-bands, the thin filaments bind the predominant intermediate filament (IF) protein in muscle, desmin, via cross-linking proteins, such as α-actinin (Figure 1). Desmin IF contribute to the mechanical and structural integrity of myofibrils by linking adjacent myofibrils laterally, and to the sarcolemma, nucleus, and mitochondria, and by promoting muscle homeostasis [13,14,15]. Loss of desmin IF during atrophy induced by fasting or denervation (i.e., loss of motor nerve input) precedes and promotes myofibril destruction [16,17,18]. Therefore, the loss of structural or regulatory components that contribute to myofibril stability (e.g., MyLCs, MyBP-C, desmin filaments) is likely to be an early event leading to myofibril breakdown.

## 3. Ubiquitin Ligases Can Act on Insoluble Filaments

The highly organized and complex structure of myofibrils is degraded upon fasting, denervation, and aging, and in many human diseases (e.g., cancer [19,20], diabetes [21,22], sepsis [23,24], and renal [25] and cardiac failure [26]), leading to loss of muscle mass and strength (i.e., atrophy). As muscles continue to contract in these various catabolic states, myofibril breakdown by the UPS must occur in a highly ordered and tightly regulated manner. Two muscle specific ubiquitin ligases, Muscle RING finger 1 (MuRF1) and Atrogin-1/MAFbx are induced in most types of atrophy, and promote proteolysis [27,28], while deficiency of either enzyme attenuates wasting [28,29]. During atrophy, Atrogin-1/MAFbx catalyzes the degradation of proteins that promote protein synthesis, whereas other ligases, in particular MuRF1 and ubiquitin tripartite motif-containing protein 32 (Trim32), catalyze the ubiquitination of myofibrillar proteins (i.e., myosin, actin, and associated proteins) and the desmin cytoskeleton [16,17,18,30,31,32]. Although ubiquitin ligases normally act on soluble substrates, recent studies surprisingly demonstrated that they can also catalyze the ubiquitination of proteins within the insoluble myofibril.

In vitro studies in muscle extracts have demonstrated that purified actin and myosin can be efficiently degraded by the proteasome [6,33], but not when these proteins are associated with each other in the actomyosin complex or intact myofibrils. These findings suggested that the UPS may not, by itself, be able to degrade components of intact myofibrils, and constituent proteins must be released by some mechanism to be substrates for degradation [6]. Consequently, several groups have suggested that proteases, such as calpains or caspases, may cleave the myofibril to accelerate disassembly before ubiquitination [34,35,36]. While this could be a possible mechanism, it has not been proven in atrophying muscles in vivo. In addition, in atrophying mouse muscles, proteolytic cleavage by calpains facilitates desmin filament loss, which is linked to myofibril destruction [17,37,38], but is not required for ubiquitination of the myofibrillar apparatus [30]. Cohen and colleagues showed that the monomeric RING E3s, MuRF1, and Trim32, are able to act directly on cytoskeletal networks and the insoluble myofibrils in vitro [16,30]. MyBP-C and MyLC2, which are important for thick filament stability, can be efficiently ubiquitinated by MuRF1 in vitro when still associated with the myofibril, and their selective loss in atrophying denervated mouse muscles in vivo precedes degradation of the major thick filament component, MyHC. Similarly, components of thin filament and Z-bands, including actin, tropomyosin, and α-actinin in intact myofibrils, as well as the desmin cytoskeleton are efficiently ubiquitinated by recombinant Trim32 [16,30], and degradation of desmin filaments during fasting or denervation precedes and promotes myofibril destruction. These findings suggest that ubiquitin ligases can ubiquitinate the proteins of intact myofibrils, and that the selective initial loss of proteins that stabilize the myofibril (such as MyLCs, MyBP-C, and desmin IF) may loosen this structure, rendering its protein constituents more susceptible for the catalytic activity of proteolytic enzymes and the proteasome. Accordingly, myofibrils isolated from 14-day denervated muscles, when myofibrillar myosin and actin are rapidly ubiquitinated and degraded [30], were more efficiently ubiquitinated by Trim32 in vitro than myofibrils from 10-day denervated muscles, in which degradation of myosin and actin was slow [18]. Thus, when desmin filaments are solubilized and protein ubiquitination is accelerated, myofibrillar constituents are more sensitive to Trim32, and probably to other ubiquitin ligases. Trim32 and certain deubiquitinating enzymes (including USP1 [39] and USP19 [40]) also promote myofibril breakdown indirectly, i.e., by reducing the major growth pathway in muscle, PI3K-Akt-FoxO signaling [41,42], resulting in increased proteolysis and decreased protein synthesis [43].

As mentioned above, in addition to its role in promoting thin filament loss, Trim32 is also responsible for the ubiquitination and the resulting degradation of desmin IF. Desmin filament phosphorylation by the protein kinase GSK3β [16,17,44] is required to enhance ubiquitination by Trim32; however, phosphorylation of Trim32′s substrates cannot be a prerequisite for ubiquitination because this enzyme can also ubiquitinate pure non-phosphorylated actin in vitro [16]. In addition, protein phosphorylation can rather protect from degradation, by preventing ubiquitination. For instance, phosphorylation of p53 upon DNA damage stabilizes this protein by reducing its association with the ubiquitin ligase, Mdm2 [45]. It has long been known that SCF ubiquitin ligases act preferentially on phosphorylated substrates (e.g., Cyclin D1, Cyclin E, β -Catenin) [5,46,47,48], a property that was considered unusual for a RING E3 such as Trim32. However, in recent years other RING E3s, in addition to Trim32, have been shown to act preferentially on phosphorylated substrates, including c-Cbl and C-terminal Hsp70-interacting protein (CHIP) [49,50].

## 4. Loss of Stabilizing Structures is a Prerequisite to Myofibril Breakdown

Desmin is a cytoskeletal type III IF protein (53 kDa) consisting of a N-terminal head, a central α-helical rod, and a C-terminal tail domains [51]. It is specifically expressed in muscle, and is critical for maintenance of tissue architecture and function. While desmin null mice are viable, they exhibit loss of lateral alignment of myofibrils and abnormal mitochondrial organization in both skeletal and cardiac muscles [52]. Mutations in desmin cause a variety of skeletal and cardiac myopathies, called desminopathies, which are characterized by toxic aggregation of mutant misfolded desmin. Consequently, myofibril function is perturbed, as well as mitochondrial homeostasis [53], leading to muscle weakness, respiratory dysfunction, and cardiomyopathies [54]. Since desmin IF are important for sarcomere stability and alignment, their initial loss during atrophy represents a key step in the process of myofibril instability and ultimate breakdown.

**Figure 2:**
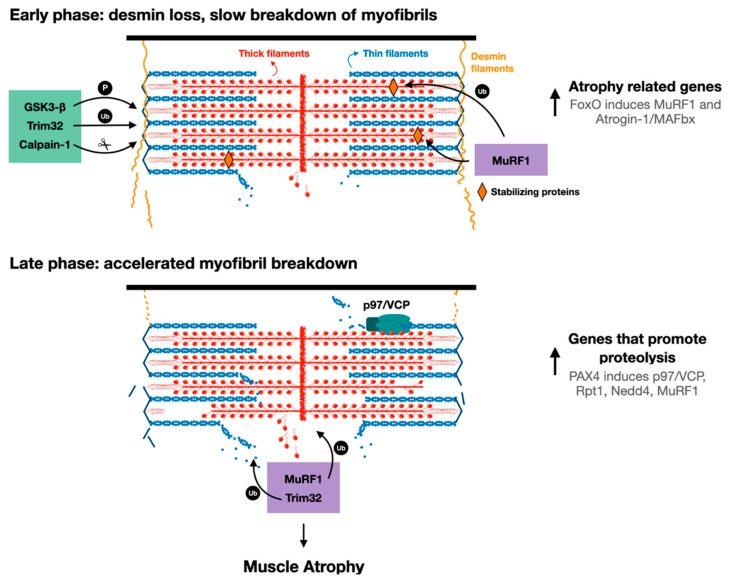
Proposed model for myofibril breakdown in atrophy. The sequence of events leading to myofibril breakdown and atrophy seems to define two major phases. In the early phase, there is a selective loss of desmin filaments and other proteins that stabilize the myofibrils, and the myofibrils are only slowly degraded. This initial loss of stabilizing systems accelerates myofibril breakdown in a more delayed phase by enzymes that promote proteolysis (e.g., UPS components such as E3s, p97/VCP), whose expression is increased by PAX4.

The sequence of cellular events leading to desmin filament loss has been recently discovered in studies on denervated mouse muscles. Early after denervation (3 days), desmin filaments are phosphorylated by the kinase GSK3-β, ubiquitinated by Trim32 (and probably other ubiquitin ligases), and then depolymerized by the Ca^2+^-dependent protease calpain-1 (Figure 2) [17,55]. These events precede significant ubiquitination and degradation of myofibrillar proteins, suggesting that the dissociation and loss of desmin filaments precede, and thus may promote, myofibril breakdown (Figure 2). In fact, introducing a dominant-negative mutant of desmin, which promotes desmin disassembly, accelerated myofibril destruction in mouse denervated muscles, indicating that loss of desmin filaments is linked to degradation of myofibrillar proteins [18]. Although desmin IF loss appears important for myofibril solubilization and proteasomal degradation, it is dispensable for myofibril ubiquitination. Accordingly, inhibition of desmin filament loss (e.g., by GSK3-β inhibition, or by downregulation of Trim32 or calpain-1) resulted in accumulation of ubiquitinated intact myofibrils in the insoluble fraction [16,17].

In denervated mouse muscles, increased phosphorylation of desmin filaments facilitated ubiquitination, but was insufficient to cause depolymerization, which occurred only 4 days later. This delay in desmin filament dissociation and loss was intriguing, and suggested that additional signals beyond phosphorylation and ubiquitination are required to promote desmin filament depolymerization and loss. Accordingly, loss of desmin filaments occurred at 7 days after denervation when cytosolic Ca^2+^ levels rose and activated calpain-1 [17]. Therefore, the timing of the elevation in Ca^2+^ levels may be rate limiting for desmin filament loss and myofibril destruction. Consistent with this idea, Ca^2+^ levels rise in different types of atrophy (e.g., fasting [17], sepsis [56,57], and cancer [58]) and in diseased muscles. For example, in *mdx* mice model for Duchenne muscular dystrophy, Ca^2+^ levels are elevated and induce protein degradation, most likely by stimulating Ca^2+^-specific proteases [59,60].

These investigations indicated that calpain-1 promotes the rapid dissociation of phosphorylated and ubiquitinated desmin filaments. A time-course analysis of desmin cleavage by calpain-1 in vitro demonstrated that desmin filaments, which were pretreated with the alkaline protein phosphatase calf intestinal (CIP), were less sensitive to cleavage by calpain-1. In addition, in atrophying muscles expressing GSK3-β dominant-negative inhibitor (a kinase-dead mutant), desmin filaments accumulated in their unmodified form, and were less efficiently cleaved by calpain-1 in vitro. Thus, calpain-1 shows a preference for phosphorylated desmin filaments [17]. The discovery that in atrophying muscles, phosphorylated and ubiquitinated desmin filaments are cleaved by calpain-1 is surprising because calpain-1 does not harbor a bona fide ubiquitin binding domain. Possibly, ubiquitination of desmin filaments facilitates depolymerization by triggering conformational changes that expose calpain-1 cleavage sites on desmin. Accordingly, two ubiquitination sites have been identified in the desmin rod domain [61], the only structured region in desmin monomers. In addition, it has been previously suggested that ubiquitination can cause conformational changes that expose calpain cleavage sites on substrate proteins [62,63]: Watkins et al. demonstrated that calpain-mediated cleavage of the regulatory domain of protein phosphatase 2A (PP2A) occurs after PP2A monoubiquitination by the ubiquitin ligase Midline-1, leading to PP2A degradation by the proteasome [55]. A similar role for ubiquitination has been demonstrated for interleukin-1 receptor, type 1 (IL1-R1): overexpression of IL1-R1 and its ubiquitin ligase, tumor necrosis factor receptor-associated factor-6 (TRAF6), in HEK293T cells led to IL1-R1 ubiquitination by TRAF6, which facilitated IL1-R1 cleavage by γ-secretase and loss [64]. Based on these findings, the authors suggested that ubiquitination-mediated cleavage could be a molecular mechanism controlling protein function and/or stability. Desmin filaments are polyubiquitinated by Trim32 in atrophying mouse muscles, and also in vitro by recombinant Trim32, and inhibition of their ubiquitination (e.g., by Trim32 downregulation) prevents depolymerization and atrophy [16,17]. This ubiquitination can also promote desmin filament depolymerization by facilitating binding of AAA-ATPase complexes, which extract ubiquitinated proteins from larger structures through ATP hydrolysis [65], and thus enhance their accessibility to the catalytic core of the proteasome. For example, the AAA-ATPase, p97/VCP, promotes myofibril disassembly during atrophy by facilitating the release of ubiquitinated myofibrillar proteins into the cytosol (see below) [18,66]. Its role (and the role of other AAA-ATPases) in promoting desmin loss in atrophy has not been investigated, and is an important question for future research.

## 5. Ubiquitinated Proteins are Released from the Myofibril by p97/VCP

The AAA-ATPase p97/VCP complex extracts ubiquitinated proteins from larger structures, and regulates various cellular processes. Mutations in p97/VCP cause inclusion body myopathy with early-onset Paget’s disease, and a late-onset frontotemporal dementia (IBMPFD) disease characterized by muscle weakness, bone deformities and weakness, and dementia [67]. While the specific mechanisms by which p97/VCP mutations cause disease are not completely understood, several studies have shown that p97/VCP mutations impair mitochondrial homeostasis [68] and ER-associated protein degradation (ERAD), probably by altering association of p97/VCP with its co-factors [69]. Studies in animal models have indicated an important role for p97/VCP in promoting muscle development and homeostasis. Loss of p97/VCP function in skeletal or cardiac muscles interferes with autophagy [70], and myofibril assembly [71,72]. In addition, knockdown of TER94, the *Drosophila* ortholog for p97/VCP, perturbs myofibril organization and function in *Drosophila* hearts, and causes cardiomyopathies [73]. Recent investigations in atrophying mouse muscles concluded that the disassembly of myofibrils also requires p97/VCP, which appears to extract ubiquitinated proteins from the myofibrillar apparatus before proteasomal degradation [18,66]. This AAA-ATPase complex is induced during atrophy by the transcription factor, paired box 4 (PAX4), when desmin filaments are lost and just before degradation of myofibrillar proteins is accelerated [18,66]. The role of p97/VCP in promoting myofibril disassembly in atrophy has been determined by in parallel analysis of the muscle insoluble (containing mainly myofibrils), and soluble, fractions using SDS-PAGE and immunoblotting with a ubiquitin antibody [18]. These analyses showed that at 14 days after denervation, when myofibrillar proteins are undergoing rapid degradation, the levels of ubiquitinated proteins are low in the insoluble fraction, and instead increase in the cytosol. However, inhibition of p97/VCP by the electroporation of a dominant negative mutant (lacking the ATPase activity) into denervated mouse muscle prevented the disassembly of ubiquitinated myofibrils, led to accumulation of ubiquitinated proteins in the insoluble fraction, and attenuated atrophy [18]. The cofactors that function together with p97/VCP in the disassembly of myofibrils in atrophy are yet to be identified. In *Caenorhabditis elegans*, myofibril assembly during muscle development requires p97/VCP and its cofactors UFD2 and CHN1 [72]; however, it is unknown if they also contribute to myofibril disassembly in atrophying muscles.

In addition to p97/VCP, other enzymes that promote ubiquitination and proteolysis are induced by PAX4 at 10 days after denervation, including the proteasomal subunit Rpt1, and the ubiquitin ligases MuRF1 and Nedd4 [18]. The major atrophy related E3s, Atrogin-1 and MuRF1, are induced earlier (i.e., at 3 days after denervation) primarily by the transcription factor FoxO [74], suggesting that there are early and late phases of the induction of genes that promote proteolysis during the debilitating process of atrophy (Figure 2). Therefore, myofibril breakdown during atrophy appears to be a two-phase process involving an initial loss of desmin filaments, which promotes the later breakdown of myofibrils. Clearly, in addition to desmin filament dissociation, an induction of p97/VCP and other catabolic enzymes in a second more delayed phase during atrophy is required to accelerate myofibril destruction (Figure 2). While distinct transcription factors appear to promote the induction of genes in these two phases (i.e., 3 days and 10 days after denervation), it remains to be determined whether they act in coordination or independently to induce genes in two phases, or to ensure that the levels of certain genes (e.g., MuRF1) remain high throughout the atrophy process.

## 6. Degradation of Myofibrillar Proteins Accompanies Systemic Disease

Myofibril breakdown occurs in various diseases including cancer cachexia, sepsis, and heart failure, and can cause a rapid loss of muscle mass. Prior investigations, primarily in animal models, have demonstrated a similar activation of proteolysis by the UPS in atrophying muscles in response to diverse catabolic stimuli, suggesting that common mechanisms promote wasting in various diseases [75]. Therefore, targeting key regulators of these common mechanisms should be beneficial for the treatment of numerous wasting conditions.

Cachexia is a severe systemic loss of muscle mass, with or without loss of fat, which is associated with many types of cancer, and often correlates with patient demise [76]. Consistently, clinical studies and recent findings in tumor bearing mice suggest that preservation of muscle mass may prolong survival [77]. The molecular mechanisms leading to myofibril breakdown and cachexia are not fully understood, although the UPS is known to play a prominent role. Studies in tumor-bearing rats demonstrated that increased levels of ubiquitinated proteins and accelerated proteolysis, primarily by the UPS, cause most of the muscle wasting and the loss of body weight [20,78,79]. Consistent with these findings, a clinical study of 23 patients diagnosed with gastric cancer at different stages, also presented loss of body weight and muscle mass, and muscle biopsies from these patients showed increased proteasome activity and ubiquitin expression compared with healthy subjects [80]. This increase in ubiquitin expression and proteasome activity has been suggested to be mediated by cytokines secreted by the tumor, such as proteolysis-inducing factor (PIF) and tumor necrosis factor-α (TNF-α). Accordingly, in vitro studies in cultured myotubes treated with PIF demonstrated increased expression of 20S and 19S subunits, and a decrease in myosin protein levels [81], while in a similar in vitro study, myotubes treated with TNF-α displayed a reduction in total protein and myosin content, and increased levels of ubiquitinated proteins [82]. Recently, findings in tumor bearing mice have uncovered a new feature of cachectic muscles, which includes plasma membrane irregularities associated with loss of the principle component of the dystrophin glycoprotein complex (DGC), dystrophin [83]. This multiprotein complex is located at discrete foci on the muscle membrane, and is vital for muscle integrity and function by linking the intracellular cytoskeleton to the extracellular matrix, hence protecting the muscle membrane from mechanical stress imposed by muscle contraction. Mutations in dystrophin cause Duchenne muscular dystrophy, and dystrophin loss in cachectic muscles leads to DGC dissociation, and correlates with induction of UPS components, and atrophy [83]. In addition, our recent findings in mouse models for atrophy indicate that, in multiple systemic catabolic states (e.g., fasting, type 2 diabetes), there is a decrease in intact DGC on the muscle membrane, which appears to precede significant loss of desmin filaments [84]. Hence, it will be important to determine whether dissociation of the DGC is a characteristic feature of atrophying muscles, and an early event leading to desmin IF loss and ultimately to myofibril breakdown.

Systemic inflammation (e.g., sepsis) also causes rapid loss of skeletal muscle mass, and activation of protein degradation by the UPS is the principal mechanism for this loss, although autophagy also plays a role [85]. Atrophying muscles from patients with sepsis or from septic rats exhibited increased proteolysis by the UPS [86] of primarily myofibrillar proteins [87,88], which was accompanied by reduced protein synthesis [89] and decreased expression of myofibrillar proteins [90]. Accordingly, incubation of septic rat muscles with proteasome inhibitors in vitro significantly blocked protein breakdown [89]. Early work by Williams et al. demonstrated increased dissociation of myofibrils in EDL muscles from septic rats, which correlated with Z-band disintegration and induction of calpain-1, calpain-2, and calpain-3/p94 [91]. Treatment with the calcium antagonist, dantrolene, attenuated myofibril disassembly [91], indicating that these Ca^2+^-dependent enzymes contribute to muscle protein breakdown in sepsis, most likely through effects on the desmin cytoskeleton [17]. Moreover, in cardiac mouse muscles, sepsis induced a similar activation of UPS components and calpains, and also caused a reduction in the protein levels of the DGC subunits, dystrophin and β-dystroglycan, and myofibrillar proteins [92,93]. This loss of intact DGC in cardiac muscle may precede overall proteolysis, as has been suggested in other types of rapid wasting [84].

Severe chronic heart failure (CHF), which often results from various cardiac disorders, impairs the ability of the cardiac ventricle to properly fill or eject blood, and leads to reduced muscle strength, muscle cross-sectional area, and exercise capacity [94]. Patients with CHF also exhibit substantial atrophy of skeletal muscles that, similar to the other diseases discussed above, exhibit increased proteolysis by the UPS [95]. Histological analyses of muscle biopsies from patients with heart failure [96] or from rat models for CHF [95] demonstrated a different pattern of atrophy: a selective loss of MyHC, especially in the diaphragm muscle. Although these pathological manifestations correlated with increased caspase-3 levels, its specific role in atrophy, and whether it catalyzes the selective loss of MyHC, remain uncertain. In addition to the selective loss of MyHC, desmin filament dissociation and breakdown also take place in the failing heart [97]. Rats subjected to aortocaval fistula (ACF), a condition that ultimately leads to heart failure, exhibited perturbations to desmin filament alignment at the Z-bands, and disorganized mitochondria. Furthermore, in ACF rat model, there was an increase in desmin phosphorylation and accumulation of desmin cleavage products in cardiac muscle, suggesting that cytoskeletal cleavage and breakdown might contribute to the progression of heart failure [97,98].

The role of the UPS in promoting various diseases has made its components attractive therapeutic targets. Several proteasome inhibitors (e.g., bortezomib or MG132) have been approved by the FDA for the treatment of hematologic cancers [99], inhibitors of specific ubiquitin ligases have been identified and entered clinical studies [100], and inhibitors of deubiquitinating enzymes have also become an intriguing field of study in recent years [101]. Injection of MG132 reduced muscle loss in cachectic tumor-bearing mice [102], in immobilized mice [103], and in zebrafish lacking the DGC component, dystrophin [104]. In addition, Bortezomib administration to golden retriever dog model for muscular dystrophy led to increased expression of certain DGC components, and alleviated muscle pathology [105]. Although these proteasome inhibitors were originally developed to treat muscle wasting [106], their use in the clinic to combat atrophy would be ill-advised because proteasome inhibition will likely alter cell composition and interfere with protein quality control. Thus, major therapeutic promise for the treatment of numerous wasting conditions will likely arise from the inhibition of key regulators of protein breakdown.

## 7. Concluding Remarks

Seminal research during the last decade has revealed that myofibrils are degraded during atrophy in a gradual, organized, and controlled manner, hence allowing muscles to continue to contract even during rapid atrophy (e.g., fasting, cancer cachexia, sepsis). It will be of interest to explore if the two-phase gene induction during atrophy (i.e., at 3 days and 10 days after denervation) and the two major catabolic events (i.e., desmin filament loss followed by myofibril destruction) characterize other more prolonged types of atrophy (e.g., aging).

The mechanisms regulating the slow turnover of myofibrils in normal muscle remain uncertain. Under normal conditions, there is probably a slow exchange of myofibrillar components with soluble precursors. Desmin filaments also likely to continuously polymerize and depolymerize in normal muscle by a mechanism requiring calpain-1, because in muscles from calpain-1 knockout mice, phosphorylated desmin accumulates as insoluble filaments [17]. These findings raise intriguing questions that merit further research about how desmin IF loss and myofibril breakdown can be accomplished in healthy or diseased muscles, without causing immediate detrimental effects on contractile function. Ubiquitin ligases and proteases can act on proteins at the surface of the myofibril to promote disassembly and degradation, and this process can be accelerated during atrophy, when the expression of such catabolic enzymes is elevated and stabilizing systems are lost. If these events during atrophy loosen the myofibrils, then proteins in the interior of the myofibril would likely be more accessible to proteolytic enzymes. Prior studies on purified filaments suggested that trituration with ATP in vitro can release “easily releasable myofilaments” (ERMs) from the surface of the myofibril [107]. However, the role of ERMs in myofibril turnover in vivo is unclear, and changes in isoform composition of myofibrillar proteins and muscle fiber type during aging cannot be explained by such a mechanism.

Substantial progress has been made in our understanding of the molecular mechanisms that mediate myofibril breakdown, and the specific role of the UPS in this process. Deciphering these mechanisms is not only important for understanding the process of filament breakdown by the UPS, but it is also important from a clinical standpoint. Identifying ubiquitin ligases, AAA-ATPases, and other UPS components that mediate desmin filament loss and myofibril breakdown is essential for the development of rational therapies to block muscle wasting and the associated disability, morbidity, and mortality.

## Data Availability

Data are available from the corresponding author on request.
